# HTLV-1 p13 Protein Hijacks Macrophage Polarization and Promotes T-Cell Recruitment

**DOI:** 10.3390/v17040471

**Published:** 2025-03-26

**Authors:** Ramona Moles, Maria Omsland, Cynthia A. Pise-Masison, Jeffrey J. Subleski, Daniel W. McVicar, Sarkis Sarkis, Anna Gutowska, Luca Schifanella, Melvin Doster, Robyn Washington-Parks, Vincenzo Ciminale, Genoveffa Franchini

**Affiliations:** 1 Animal Models and Retroviral Vaccines Section, Vaccine Branch, National Cancer Institute, National Institutes of Health, Bethesda, MD 20892, USAmasisonc@mail.nih.gov (C.A.P.-M.);; 2 Department of Cell and Molecular Biology, Center for Immunology and Microbial Research, Cancer Center and Research Institute, University of Mississippi Medical Center, Jackson, MS 39216, USA; 3 Department of Safety, Chemistry, and Biomedical Laboratory Sciences, Western Norway University of Applied Science, 5063 Bergen, Norway; 4 Cancer and Innovation Laboratory, Center for Cancer Research, National Cancer Institute, Frederick, MD 21702, USA; 5 Department of Surgery, Oncology and Gastroenterology, University of Padua, 35122 Padua, Italy; 6 Veneto Institute of Oncology IOV—IRCCS, 35128 Padua, Italy

**Keywords:** HTLV-1, p13, viral protein, mitochondria, monocytes, macrophages, cytokines, adult-T-cell leukemia/lymphoma, ATLL, HTLV-1 associated myelopathy/tropical spastic paraparesis, HAM/TSP

## Abstract

The human T-cell leukemia type-1 (HTLV-1) retrovirus establishes chronic life-long infection in a fraction of infected individuals associated with severe pathological conditions. Although the mechanism driving disease development is not fully understood, current evidence indicates the essential functions of viral regulatory proteins. Among these, the p13 protein has previously been shown to localize to the inner mitochondrial membrane in T cells, altering mitochondrial biology and T-cell function. While CD4^+^ T cells are the primary cell target of HTLV-1 infection, genomic viral DNA has also been detected in monocytes, macrophages, and dendritic cells, which orchestrate innate and adaptive immunity and play a critical role in protecting against virus-induce diseases by establishing the appropriate balance of pro and anti-inflammatory responses. Given the central role of mitochondria in monocyte differentiation, we investigated the effect of p13 in monocytes/macrophages and found that by localizing to mitochondria, p13 affects mitochondrial respiration. Moreover, we demonstrate that p13 expression affects macrophage polarization to favor the recruitment of CD4^+^ T cells, the primary target of the virus, potentially facilitating the spread of viral infection and the development of disease.

## 1. Introduction

The human T-cell leukemia virus type 1 (HTLV-1) retrovirus is responsible for pathological conditions, including cancer and neurodegenerative and inflammatory diseases. Approximately 2–5% of infected individuals develop adult T-cell leukemia (ATL), HTLV-1-associated myelopathy/tropical spastic paraparesis (HAM/TSP), and other frequencies of immune-based pathologies such as infectious dermatitis, arthropathies, uveitis, and Sjögren’s syndrome. A common feature of HTLV-1 infection is immune dysregulation with high levels of both pro- and anti-inflammatory cytokines, suggesting a continuous functional activation of the immune system. Nevertheless, HTLV-1 infection is a lifelong condition in which the virus cannot be eradicated [1,2,3,4,5,6,7]. The viral mechanisms that allow HTLV-1 persistence in the host despite strong innate and adaptive immune responses are yet to be fully understood.

By alternative splicing, the HTLV-1 genome encodes for different viral proteins that modulate cell growth and immune responses [8,9]. From a single-spliced mRNA, the p13 protein is encoded from open reading frame II (orf-II) [10]. In T cells, p13 expression targets the inner mitochondrial membrane and increases mitochondrial K^+^ permeability and mitochondrial ROS [11,12]. These changes affect cell turnover, activating primary T-cells and reducing the proliferation of transformed cells and their sensitization to apoptosis [13]. In the presence of HTLV-1 viral protein Tax, which transactivates the viral promoter, p13 undergoes ubiquitylation and partly relocates to the nucleus. Nuclear p13 binds Tax and inhibits its transcriptional activity [14]. These findings suggest that the protein might exert distinct functions depending on its intracellular localization, affecting the turnover of infected cells and the balance between viral latency and productive infection.

T cells are the primary target of HTLV-1, in which the virus integrates and establishes a life-long viral reservoir [15,16,17,18,19,20,21,22,23]. However, the HTLV-1 genome is also detected in monocytes and dendritic cells in patients in vivo [24,25,26,27,28]. Moreover, all monocyte subsets (classical, intermediate, and non-classical) carry viral DNA, contributing up to 15% of the total virus burden [24,25,26,27,28], suggesting their importance in HTLV-1 infection and pathogenesis. As part of innate immunity, monocytes and their derived macrophages serve three key functions: phagocytosis, antigen presentation, and cytokine production. After their release from the bone marrow, monocytes circulate in the bloodstream for about 1–3 days before migrating into tissues, where they differentiate into macrophages [29]. Monocytes and macrophages are central for eliminating pathogens and orchestrating inflammation [30]. They play an essential role in generating inflammatory mediators, activating innate and adaptive immunity in response to viral infection, and contributing to the resolution of inflammation and tissue homeostasis [31,32]. Monocytes/macrophages are particularly plastic, with the ability to acquire different phenotypes depending on the tissue type and environmental stimuli, and they play diverse roles in the inflammatory response. During viral infection, monocytes are recruited to the site of inflammation, where they ultimately differentiate into inflammatory macrophages. The pro-inflammatory state is crucial in eliminating pathogens, and the switch from a pro-inflammatory to an anti-inflammatory/pro-resolving phenotype promotes the resolution of inflammation and maintains homeostasis [33].

While extensive studies have been done to investigate the role of p13 in T-cell function and proliferation, its role in myeloid function, differentiation, and proliferation has not been studied [34]. Because it has previously been shown that p13 influences ROS and signal transduction pathways, which are known to impact monocyte function [35], we hypothesized that p13 alters monocyte biology to influence viral infections, persistence, and spread. Using a monocytic cell line continuously expressing p13 (THP-p13), we found that, while not influencing cell turnover, p13 does affect mitochondria respiration and macrophage differentiation in response to stimuli and alters cytokine production. Furthermore, these changes create a microenvironment that favors the recruitment of immune cells, potentially facilitating virus spreading and the development of disease. These findings have significant implications for our understanding of immune responses in the context of HTLV-1 and are crucial for the development of effective immunotherapy for patients.

## 2. Materials and Methods

### 2.1. Plasmid and Cell Lines

The p13-HA-GFP construct encodes the mammalian codon-optimized p13 (with a modification in amino acid number 13 from glutamic acid [E] to glycine [G]) in-frame with an HA and GFP tag in a retroviral pBABE-puro vector. pBABE-puro was kindly provided by Dr. Hartmut Land, Dr. Jay Morgenstern, and Dr. Robert Weinberg (Addgene plasmid # 1764; https://www.addgene.org/1764/ [accessed on 24 March 2025]; RRID: Addgene_1764) [36]. The p13 sequence fused with HA and GFP was cloned into pBABE-puro using the primers BamHI-p13-F 5′-AAAGGATCCGCCACCATGCTGA-3′ and p13-SalI-R 5′-AAAGTCGACTTACTTGTACAGCTCGTCC-3′. All constructs were verified by sequencing.

THP-1 cells (ATCC; TIB-202) were grown in RPMI-1640 complete medium (supplemented with 1% penicillin/streptomycin and 10% heat-inactivated FBS). Transduction of the cells was performed as previously described [37] but using pUMVC (9 µg) instead of ps-PAX-2 [38]. Cells transduced with p13-HA-GFP or an empty vector were grown in 1.67 µg/mL puromycin for 5 days until resistance was obtained. The p13-HA-GFP cells were sorted by GFP expression giving 98% GFP-positive cells. HeLa cells (ATCC; CCL-2) were grown in Dulbecco’s Modified Eagle Medium (DMEM) complete medium (supplemented with 1% penicillin/streptomycin and 10% heat-inactivated FBS). HeLa cells were transfected with p13-HA-GFP or empty vector using Lipofectamine 3000 (Invitrogen, Waltham, MA, USA), according to the manufacturer’s instructions. Following transfection, cells were grown in puromycin for 5 days until resistance was obtained. The p13-HA-GFP cells were sorted by GFP expression, resulting in >95% GFP-positive cells. THP-pBABE (THP-Ctrl), THP-p13-HA-GFP (THP-p13), and the HTLV-1A-producing B cell line, the 729-HTLV-1 WT cells were maintained in RPMI-1640 complete medium (supplemented with 1% penicillin/streptomycin and 10% heat-inactivated FBS).

### 2.2. Immunoblotting

A total of 1–2 × 10^6^ cells were washed with PBS and lysed with RIPA buffer following incubation on ice for 10 min before centrifugation for 15 min at 12,000 RPM. Protein concentration was determined using Bradford assay (BioRad), and typically, 20–30 µg of protein was loaded onto the gel. Electrophoresis was performed at 110 V for 1 h 30 min. Transfer blotting was performed for one hour at 150 mA. Blocking was performed for 30 min to 1 h in 5% dry milk in PBS-Tween or 5% BSA in PBS-Tween, HA (1:1000), GFP (1:1000), and β-actin (1:1000) (Cell Signaling Technologies, St. Louis, MO, USA). All primary antibodies were incubated overnight at 4 °C diluted in 1% dry milk in PBS-TWEEN with secondary antibody HRP-goat-anti-mouse/rabbit (diluted 1:10 000; GE Healthcare, Chicago, IL, USA).

### 2.3. Immunofluorescence

Sixty thousand cells were cytospun (Cytospin 2, Shandon) on each coverslip and fixed with 4% PFA for 15 min at room temperature in the dark, followed by a quick wash in PBS1x. Permeabilization was performed with ice-cold methanol, and coverslips were stored at methanol at −20 °C for at least 20 min. Blocking was performed for 30 min with 30 µL droplets of 0.5% BSA in PBS1x and incubation with 30 µL droplets of primary antibody diluted in blocking solution at 4 °C overnight. Cells were investigated with LSM 510 confocal microscope with alpha Plan-Apochromat 63x/1.40 oil objective using the following laser sets: Argon (Ar)—30 mW/488 and Helium/Neon I (HeNeI)—1 mW/543 used Plan-Apochromat 63x, 1.40 NA Zeiss objective, GaASP detectors (Alexa 594), and PMT (DAPI). Figures were created using the orthogonal view option in Fiji software v. 2.14.0/1.54f.

### 2.4. Proliferation and Viability Assay

Following the manufacturer’s instructions, p13-expressing cells and Ctrl cells were stained with CellTrace™ Far Red in the dark for 30 min at 37 °C. THP-Ctrl and p13-expressing cells were treated with increased Staurosporine concentrations (0.01, 0.1, 1 μM). The MFI of CellTrace™ Far Red was measured by flow cytometry every 24 h for three days. Cells were additionally stained with viability dye (Live/Dead Fixable Blue dye; ThermoFisher Scientific, Eugene, OR, USA) to assess the percentage of live cells. Following the manufacturer’s instructions, cells were stained in the dark for 30 min at room temperature. The percentage of live cells was measured by flow cytometry at 0, 24, 48 and 72 h. Data were analyzed using FlowJo Version 10.6.

### 2.5. Seahorse Metabolic Flux Analysis

The oxygen consumption rate (OCR) and extracellular acidification rate (ECAR) of THP cell lines were analyzed using a Seahorse XF-96e Analyzer (Agilent Technologies, Santa Clara, CA, USA). For THP cells, 100,000 cells were resuspended in RPMI-based Seahorse media pH 7.4 (Agilent) with added glucose (5 mM) and L-glutamine (1 mM). Cells were plated in an XF-96e microplate and incubated for 1 h in an atmospheric incubator at 37 °C before analysis. The following metabolic modulators purchased from Sigma (St. Louis, MO, USA) were used during OCR and ECAR measurements of THP-1, respectively, including etomoxir (5 µM or 10 µM), oligomycin (0.6 µM or 1 µM), FCCP (0.7 µM or 1 µM), antimycin (0.5 µM or 1 µM), and rotenone (0.05 µM or 1 µM).

### 2.6. Efferocytosis Assay

THP-p13 and THP-Ctrl cells were labeled with CytoTell Blue (Cayman Chemical, Ann Arbor, MI, USA) according to the manufacturer’s instructions. Briefly, cells were counted and suspended at a cellular density of 1 × 10^7^ cells/mL and incubated with an equal amount of 2X CytoTell Blue at 37 °C for 30 min in the dark. Cells were washed twice with media (RPMI 10% FBS) to remove excess dye. According to the manufacturer, effector cells (729 HTLV-1 WT cells; HTLV-1 producing B cell line [39]) were stained CellTrace Far Red Cell (ThermoFisher Scientific, Eugene, OR, USA). Target cells were treated with 1 μM of Staurosporine for 3 h. The effector cells were washed and treated with PMA (200 nM) overnight, and target cells were added to the well with (ratio 1:1) and left for 2, 4, 8, and 18 h. The 10^6^ cells were kept in a separate well without THP-1 for compensation and as a gating control. Cells were then collected, and samples were analyzed by flow cytometry. Data were analyzed using FlowJo Version 10.6. For confocal imaging, THP-Ctrl cells well grown on 35 mm, No. 1.5 Coverslip, and a 14 mm Glass Diameter Uncoated Dish (Mattek-A Bico Company, Ashland, MA, USA) pretreated with Poly-D-Lysine (ThermoFisher Scientific, Eugene, OR, USA). Cells were labeled with CytoTell Blue and treated with PMA as described above. 729 HTLV-1 WT-Staurosporine-treated cells labeled with Far Red were added to the dish (ratio 1:1). Following 2 h of incubation, the live cells were imaged with an A1R Laser Scanning Confocal microscope (Nikon, Melville, NY, USA) using a 60x oil immersion objective. The Z-series image stacks (0.3 micrometers apart) were acquired to verify the engulfment of Staurosporine-treated. The images were collected using NIS-Elements imaging software v. 6.10.01 (Nikon, Melville, NY, USA) and adjusted for brightness and contrast using Fiji software.

### 2.7. M1 and M2 Macrophage Differentiation

THP-p13 and THP-Ctrl cells were plated at a cellular density of 10^6^ cells/mL. Cells were treated with PMA at a final concentration of 10 ng/mL. After 24 h, the culture medium was removed from each well and washed gently with the medium. Appropriate stimulation was added for 48 h. M1 stimuli: LPS 15 ng/mL (Sigma 1 mg; P1585-1 mg) and IFN-γ 50 ng/mL (PeproTech, Cranbury, NJ, USA; 300-02-250 μg). M2 stimuli: IL-4 25 ng/mL (PeproTech; 200-04-100 μg) and IL-13 25 ng/mL (PeproTech; 200-13-100 μg). The THP-p13 and -Ctrl were stained with anti-human CD14, CD16, CD80, CD86, CD163, and CD206 together with a viability dye for 30 min at room temperature in the dark. The cells were then collected and measured using flow cytometry to analyze the samples. Data were analyzed using FlowJo Version 10.6.

### 2.8. Measuring Mitochondrial ROS

Following M1 and M2 differentiation, the THP-p13 and THP-Ctrl cells were stained with the ROS indicator MitoSOX Red according to the manufacturer’s instructions (Invitrogen, MitoSOX™ Red) and measured by flow cytometry.

### 2.9. Luminex

Cryopreserved supernatants were analyzed using Multiplex assays (EMD Millipore Corporation, Billerica, MD, USA). The samples were assayed following the manufacturer’s instructions. Cellular supernatant was assayed for the following cytokines: IL-1β, IL-6, IL-8, IL-10, IL-12 (p40), IL-12 (p70), IL-18, and TNF-α. Briefly, the samples were thawed on ice, and 25 µL of cellular supernatant was loaded singly onto the plate and mixed with 25 µL of assay buffer and 25 µL of magnetic beads. The plates were incubated at 4 °C for 18 h under agitation at 650 rpm. Following incubation, the plate was washed, 25 µL of detection antibody was added to each well, and incubated for 1 h at room temperature. An amount of 25 µL of Streptavidin-PE was then added to each well and incubated for 30 min at room temperature. Finally, the plate was washed, and 150 µL of sheath fluid was added to each well. The samples were acquired on a Bio-Plex^®^ 200 System (Bio-Rad, Hercules, CA, USA).

### 2.10. Migration Assay

A trans-well migration assay was applied to study the migration behavior of human PBMCs. Trans-well inserts with pore sizes of 3 μm were employed. PBMCs of three different donors were loaded in 400 μL of serum-free RPMI 1640 into the upper chamber of the trans-well insert. The supernatant (400 μL) from THP-Ctrl or THP-p13 cells following M1 stimulation was added to the lower chamber. The cells were then allowed to migrate for 12 h. The cells that migrated across the membrane were counted at 2 or 12 h using an inverted microscope.

### 2.11. Statistical Analysis

Statistical significance was verified with an unpaired two-tailed Student’s *t*-test. The *p*-values are summarized with asterisks, * (*p* ≤ 0.05), ** (*p* ≤ 0.01), *** (*p* ≤ 0.001), and **** (*p* ≤ 0.0001).

## 3. Results

### 3.1. p13 Localizes to Mitochondria and Changes Monocyte Metabolism

**Figure 1:**
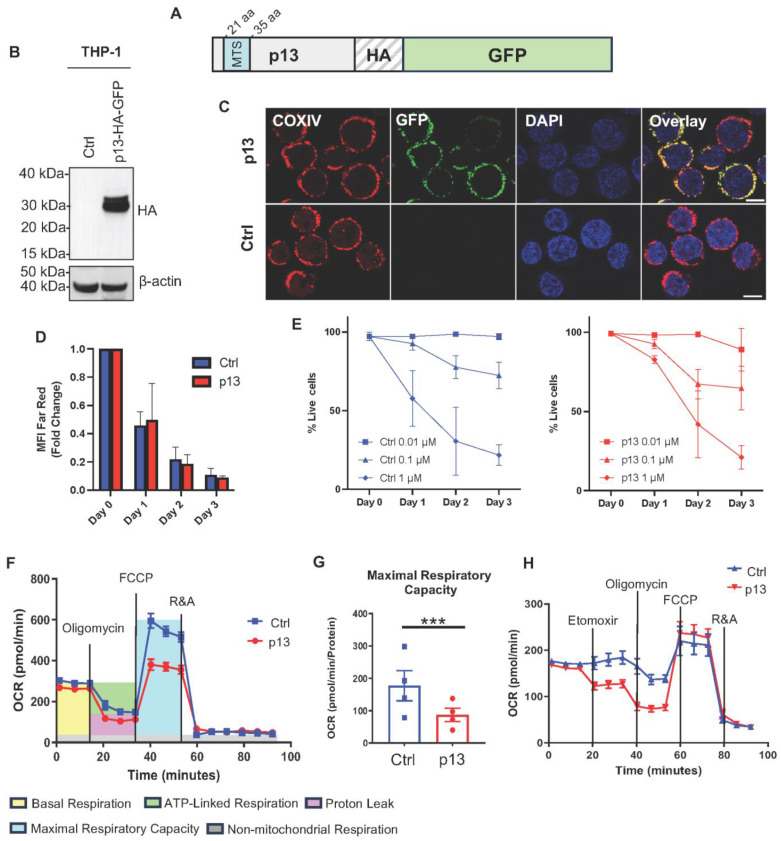
P13 targets mitochondria in monocytic cells. (**A**) Schematic representation of p13-HA-GFP viral protein. The p13 protein fused to HA and GFP carboxy terminal tags was cloned in a retroviral vector pBABE. The N-terminal region of p13 has a mitochondrial targeting sequence (MTS) from amino acids 21 to 35. (**B**) Immunoblot for HA expression from total cellular extracts of THP-1 cells transduced with retrovirus expressing p13-HA-GFP. Cells transduced with empty retrovirus were used as control (Ctrl). β-Actin expression was used as a loading control. (**C**) Stable cell lines THP-Ctrl or THP-p13 were adhered to glass slides by cytospin and stained with an antibody to complex IV (COXIV). Scale bar: 10 µm. (**D**) Proliferation assay of THP-Ctrl or THP-p13. Cells were stained with CellTrace™ Far Red, and MFI was measured by flow cytometry every 24 h for three days. The results of three independent experiments were graphed; Ctrl is labeled in blue, and p13-expressing cells in red. Statistical significance was verified with a Student’s *t*-test. No statistical significance was noted between p13-expressing cells and Ctrl. (**E**) THP-Ctrl and THP-p13 cells following the treatment with increased concentration of Staurosporine (0.01, 0.1, 1 μM). Cells were stained with Live/Dead Fixable Blue dye (ThermoFisher Scientific) to measure the percentage of live cells every 24 h for three days by flow cytometry. The results of three independent experiments were graphed; Ctrl and p13 cells are labeled in blue and red, respectively. Statistical significance was verified with a Student’s *t*-test. No statistical significance was noted between p13-expressing cells and Ctrl. (**F**) Seahorse extracellular flux analysis measured the oxygen consumption rate in THP-p13 or THP-Ctrl cells. A representative rate of spare respiratory capacity is shown. Different mitochondrial parameters are as follows: basal respiration (yellow), ATP-linked respiration (green), proton leak (magenta), maximal respiratory capacity (blue), and non-mitochondrial respiration (gray). (**G**) The maximal respiratory capacity rate was graphed for THP-Ctrl (blue) and THP-p13 (red). Statistical significance was verified with a Student’s *t*-test and reported in the figure. The *p*-values are summarized with asterisks, *** (*p* ≤ 0.001). (**H**) Seahorse extracellular flux analysis measured the oxygen consumption rate in p13-expressing and control THP cells.

To investigate the role of p13 in monocytes and macrophages, we used the human monocytic THP-1 cell line as a cellular model [40]. We transduced THP-1 cells with the pBABE retroviral vector empty (THP-Ctrl) or expressing p13 tagged at the C-terminus with hemagglutinin (HA), Green Fluorescent Protein (GFP), and THP-p13-HA-GFP (hereafter THP-p13; Figure 1A). After puromycin selection of stable clones, transduced cells were sorted to isolate a uniform GFP-positive cell population. We confirmed the stable expression of the 30 Kilo Dalton (kDa) p13-HA-GFP fusion protein by Western blot analysis (Figure 1B).

In T cells, p13 localizes in the inner membrane of the mitochondria [14,41,42] due to 10 amino acids in the mitochondrial targeting sequence (MTS) at the N-terminus of the protein (amino acids 21–35; Figure 1A). We therefore investigated the cellular localization of p13 in monocytic cells by confocal microscopy. We examined p13 subcellular distribution and localization relative to the mitochondrial resident protein Cytochrome C oxidase IV (COXIV), a well-established inner membrane mitochondria marker [43]. As shown for other cell types [44], our data show that p13 primarily co-localizes with COXIV in the mitochondria (Figure 1C) in THP-1, as well as in HeLa cells (Figure S1A,B).

Since p13 has been shown to reduce virus proliferation in T cells [41], we measured the effect of p13 expression on monocyte growth. We measured proliferation by staining THP-p13 and THP-Ctrl cells with CellTrace™ Far Red, an intracellular dye that undergoes dilution in daughter cells following cell division and whose decreasing fluorescence over time allows for visualization and quantification. Using FACS analysis, we measured the level of cellular fluorescence every 24 h for three days (Figure 1D and Figure S2A). Our data show that both THP-Ctrl and THP-p13 cells divide at a similar rate, and no statistical significance was noted at any time point (Figure 1D).

Next, we investigated if p13-expressing cells are more susceptible to cell death since the viral protein is known to induce apoptosis in T cells [45]. We exposed THP-Ctrl and THP-p13 cells to logarithmically increasing concentrations of Staurosporine (0.01, 0.1, 1 μM). Staurosporine induces rapid and prolonged intracellular free calcium levels (Ca^2+^), accumulation of mitochondrial reactive oxygen species (ROS), and subsequent mitochondrial dysfunction [46]. We first measured the antiproliferative effect of Staurosporine by monitoring cell division with CellTrace™ Far Red. The antiproliferative effect of Staurosporin in both THP-p13 and THP-Ctrl was minimal at the concentration of 0.01 μM (Figure S2B,C). However, both 0.1 and 1 μM concentrations of Staurosporine blocked cell division 24 h following treatment (Figure S2B,C). As expected, these higher concentrations of Staurosporine induced cell death as measured using a viability dye (Figure 1E). Similarly, no change in cell death induction was noted on days 1, 2, or 3 following treatment with increased concentrations of Staurosporine (Figure 1E and Figure S3A,B). Overall, our data show that p13 does not alter monocyte proliferation or cell death following treatment with Staurosporine.

Since p13 localizes in the mitochondria in monocytic cells, we investigated its possible effect on mitochondrial functions. We used the Seahorse assay to measure the oxygen consumption rate (OCR) in response to modulators of key components of the mitochondrial electron transport chain (ETC). We treated THP-Ctrl and THP-p13 cells with inhibitors and measured the OCR in real time to study different mitochondrial respiration parameters. In the Seahorse assay, the basal respiration (Figure 1F, in yellow) is calculated by subtracting the OCR of baseline from the non-mitochondrial respiration (Figure 1F, in grey). Oligomycin, an inhibitor of the mitochondrial complex V (ATPase), is added to the culture and used to calculate ATP-linked respiration (Figure 1F, in green) by subtracting the oligomycin OCR from the basal respiration rate. The proton leak respiration (Figure 1F, in magenta) is instead calculated by subtracting non-mitochondrial respiration from the oligomycin OCR. Next, the carbonyl cyanide-p-trifluoromethoxy-phenyl-hydrazon (FCCP), a protonophore, is added to the culture. This compound disrupts ATP synthesis by transporting protons across the mitochondrial inner membrane, interfering with the proton gradient. The collapse of the inner membrane gradient allows the ETC to function at its maximal rate. The maximal respiratory capacity (Figure 1F, in turquoise) is calculated by subtracting non-mitochondrial respiration from the FCCP rate. Finally, rotenone and antimycin A (R&A), inhibitors of complex I and III, respectively, are added to disrupt the ETC function. The resulting rate reveals non-mitochondrial respiration. In addition, no differences in basal respiration or ATP production (Supplementary Figure S4A,B) were noted. Our data does show that p13-expressing cells have lower maximal respiration capacity compared to the control (Figure 1F,G), and thus a drop in spare respiratory capacity (the difference between the amount of ATP a cell produces at rest and at maximum), consistent with p13 increasing potassium influx [12].

Mitochondria can use different sources of fuel for energy production. Glutamine and glucose are oxidized in the mitochondria to supply the most usable energy for cellular function. We decided to test the dependency of mitochondrial respiration of p13-expressing cells from glucose and glutamine. Depletion of glucose or glutamine demonstrates that p13-expressing cells utilize glutamine normally and are minimally affected by glucose deprivation (Figure 1H). However, we noted that respiration capacity was not restored to normal levels when the transfer of acyl-carnitines into the mitochondria was blocked by treating the THP-p13 cells with Etomoxir (ETO), an inhibitor of fatty acid oxidation (FAO) (Figure 1H, Supplementary Figure S4C). Our data suggest that p13 increases the energetic cell dependency on fatty acid oxidation rather than glucose utilization (Figure 1H). Overall, we demonstrate that the HTLV-1 p13 protein targets mitochondria and reprograms mitochondrial metabolism in monocytes.

### 3.2. The Effect of p13 Expression on Efferocytosis and Differentiation

**Figure 2:**
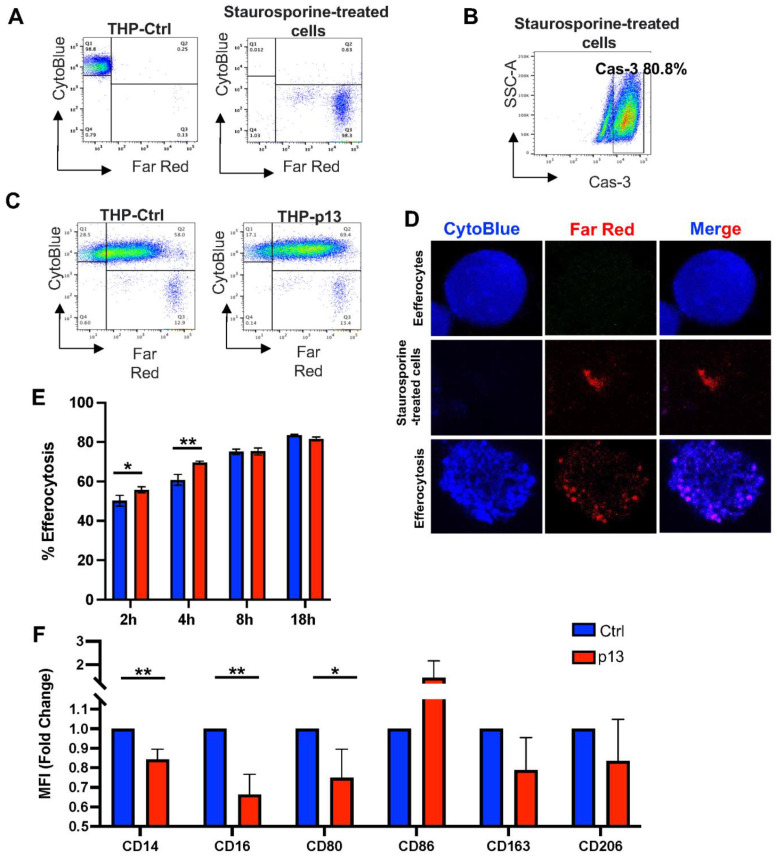
P13 affects efferocytosis and macrophage differentiation. (**A**) The THP-p13 (red) and THP-Ctrl (blue) cells were labeled with CytoTell Blue according to the manufacturer’s instructions and treated with PMA to induce maturation (**left**). Target cells, 729 HTLV-1 WT, were labeled with Far Red and treated with 1 μM of Staurosporine for 3 h (**middle**). (**B**) Cell death of Staurosporine-treated cells was measured by straining cells with Caspase 3. (**C**) Target cells were added to the well at an effector to target cell ratio of 1:1 and left for 2, 4, 8, and 18 h. Efferocytosis was measured by gating singlets and the double CytoTell Blue and Far Red positive cells. (**D**) Following 2 h co-cultivation, THP cells labeled with CytoTell Blue and Staurosporine-treated cells (Far Red) were imaged to confirm cell engulfment. (**E**) Results of three independent experiments were graphed; Ctrl and p13-expressing cells are labeled in blue and red, respectively. (**F**) Following PMA treatment, THP-p13 and THP-Ctrl cells were collected and stained for CD14, CD16, CD80, CD86, CD163, and CD206 surface markers and viability dye. Cells were gated by size, singled, and live. For THP-p13, cells were additionally gated for GFP. The MFI of each marker was graphed as a fold change compared to the PMA-treated control cells. The results of three independent experiments are shown. (**E**,**F**) Statistical significance was verified with a Student’s *t*-test and reported in the figure. *p*-values are summarized with asterisks, * (*p* ≤ 0.05), ** (*p* ≤ 0.01).

During viral infection, efferocytosis clears dead cells and maintains tissue homeostasis [47]. The presence of dead cells recruits phagocytes, which rapidly engulf and digest their cargo to avoid necrosis and proinflammatory cytokine release. Mitochondrial respiration is crucial to phagocyte functions, including efferocytosis [48,49]. To investigate the possible role of p13 in this process, we compared the phagocytic ability of THP-Ctrl and THP-p13 cells to engulf Staurosporine-treated HTLV-1 infected cells (Figure 2). We stained phagocytic THP-Ctrl and THP-p13 cells with CytoBlue (Figure 2A, left) and labeled Staurosporine-treated HTLV-1 target cells with Far Red (Figure 2A, right). Staurosporine treatment resulted in more than 80% cell death, as indicated by intracellular active Caspase-3 staining (Figure 2B). THP-Ctrl or THP-p13 cells were cultured with equivalent numbers of Staurosporine-treated cells at 2, 4, 8, and 18 h, non-engulfed cells were removed, and efferocytosis was measured by flow cytometry. Engulfed cells were measured by gating on singlets first to exclude cell complexes and then CytoBlue and Far Red double-positive cells (Figure 2C). To further confirm cell engulfment, Z-series image stacks were acquired with confocal microscopy to verify the internalization and degradation of Staurosporine-treated cells (Figure 2D). Staurosporine-treated cells stained in Far Red were clearly detected in the cytoplasm of efferocytes (Figure 2D, lower panels). A higher percentage of engulfed cells was observed in p13 cultures at both 2 and 4 h following incubation but was not maintained thereafter (Figure 2E), suggesting a saturation effect. Overall, our data showed that p13-expressing cells engulfed Staurosporine-treated cells more efficiently than control cells early after co-culture but not at later time points.

Because THP-Ctrl and THP-p13 cells were treated with phorbol myristate acetate (PMA) to induce macrophage-like differentiation for the above efferocytosis assay [50], we evaluated macrophage-specific markers to test the effect of p13 expression on monocyte differentiation. Our analysis showed that p13 significantly reduced CD14, CD16, and CD80 cell surface expression in response to PMA, suggesting impairment of macrophage differentiation (Figure 2D). No change in CD86, CD163, or CD206 surface markers was noted (Figure 2D) after PMA stimulation, consistent with the ability to engulf Staurosporine-treated cells.

### 3.3. p13 Influences M1 and M2 Macrophage Polarization

During both homeostasis and viral infection, circulating monocytes leave the bloodstream and migrate into tissues where local stimuli such as growth factors, cytokines, and viral products cause them to differentiate into macrophages. Recruitment and monocyte differentiation are essential for effectively controlling and clearing viral infections. Many viruses directly or indirectly influence monocyte differentiation to macrophages to favor viral persistence and pathogenesis [51,52]. Macrophages that have high plasticity can adopt two major polarization states with stimulation: the classically activated type 1 (M1) and the alternatively activated type 2 (M2). M1 macrophages are classically activated by the bacterial cell wall components that compose lipopolysaccharide (LPS). In addition, M1-polarized macrophages play critical roles in antiviral responses by the release of proinflammatory cytokines. M2 macrophages are instead involved in tissue remodeling and repair [31,53].

**Figure 3:**
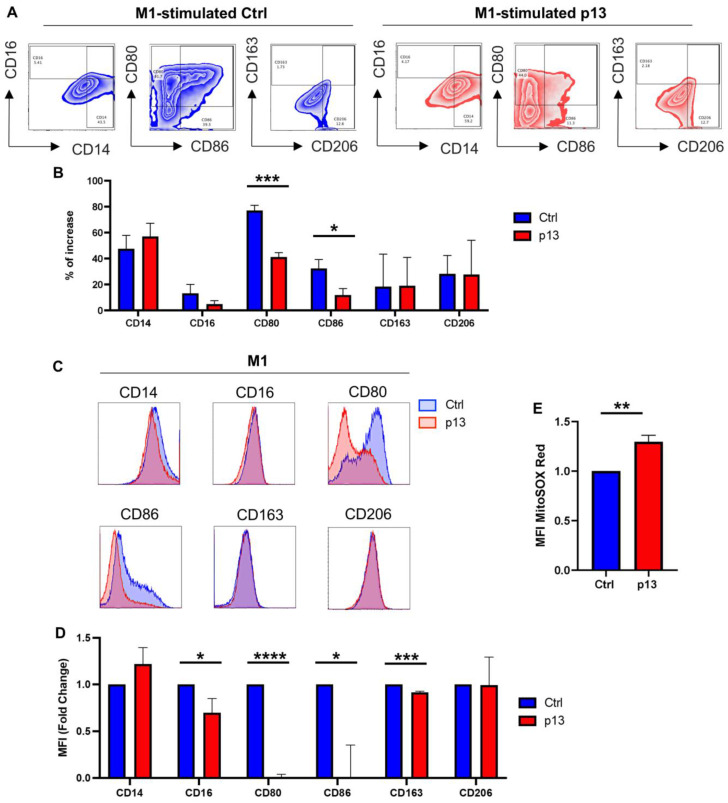
Role of p13 in M1 macrophage polarization. (**A**) THP-p13 and THP-Ctrl cells were treated with PMA at a final concentration of 10 ng/mL for 24 h. Cells were washed gently with medium, and appropriate stimulation was added for 48 h. M1 stimuli: LPS 15 ng/mL and IFN-γ 50 ng/mL. Following 48 h, cells were collected and stained for CD14, CD16, CD80, CD86, CD163, and CD206 surface markers and viability dye. Cells were gated by size, singled, and live. For THP-p13, cells were additionally gated for GFP. The percentage of increased CD14 and CD16; CD80 and CD86; CD163 and CD206 positive cells following stimulation was graphed. (**B**) The percentage of increased frequency of CD14, CD16, CD80, CD86, CD163, and CD206 compared to untreated THP-Ctrl was graphed. The results of three independent experiments are shown; Ctrl and p13-expressing cells are labeled in blue and red, respectively. (**C**) Representative mean fluorescence intensity (MFI) histograms for CD14, CD16, CD80, CD86, CD163, and CD206 surface markers are shown. THP-Ctrl and THP-p13 cells are colored in blue and red, respectively. (**D**) MFI of CD14, CD16, CD80, CD86, CD163, and CD206 surface markers of THP-p13 was graphed as a fold change compared to THP-Ctrl. The results of three independent experiments are shown; Ctrl is labeled in blue and p13-expressing cells are labeled in red. (**E**) MitoSOX-based flow cytometry with Red fluorescence was used to measure mitochondrial ROS formation in p13 M1 differentiated cells compared to the M1 differentiated control. The results of three independent experiments were graphed as a fold change. (**B**,**D**,**E**) Statistical significance was verified with a Student’s *t*-test and reported in the figure. *p*-values are summarized with asterisks, * (*p* ≤ 0.05), ** (*p* ≤ 0.01), *** (*p* ≤ 0.001), and **** (*p* ≤ 0.0001).

To achieve M1 polarization, we treated THP-p13 and THP-Ctrl cells with PMA for 24 h and then stimulated the cells with LPS and IFN-γ as previously described [54]. Phenotypically, M1 macrophages express high levels of the co-stimulatory molecules CD80 and CD86. As expected, following stimulation, surface expression of CD80 and CD86 dramatically increased on treated THP-1 cells compared to the untreated control cells (Figure S5A,B, magenta). To test if p13 affects M1 differentiation, we compared the expression of CD14, CD16, CD80, CD86, CD163, and CD206 specific markers following stimulation in THP-p13 compared to THP-Ctrl cells (Figure 3A,B). Changes in receptor expression following M1 (PMA/LPS/IFN-γ) stimulation revealed an increase in the percentage of cells expressing all molecules examined. However, a significant decrease in the induction of cells with increased CD80 and CD86 expression in THP-p13 compared to control cells was also observed (Figure 3B). In addition, when we compared normalized MFI to examine expression levels for each surface marker, we detected significant decreases in CD16, CD80, CD86, and CD163 (Figure 3C,D). Since mitochondrial ROS activation promotes M1 macrophage differentiation [55], we used flow cytometry to measure the level of MitoSOX-positive cells (an indicator of ROS) after PMA/LPS/IFN-γ stimulation. Surprisingly, following M1 differentiation, p13-expressing cells had increased levels of mitochondrial ROS compared to THP-Ctrl cells (Figure 3E). Together, these results indicate that p13 disrupts M1 macrophage differentiation of monocytes.

**Figure 4:**
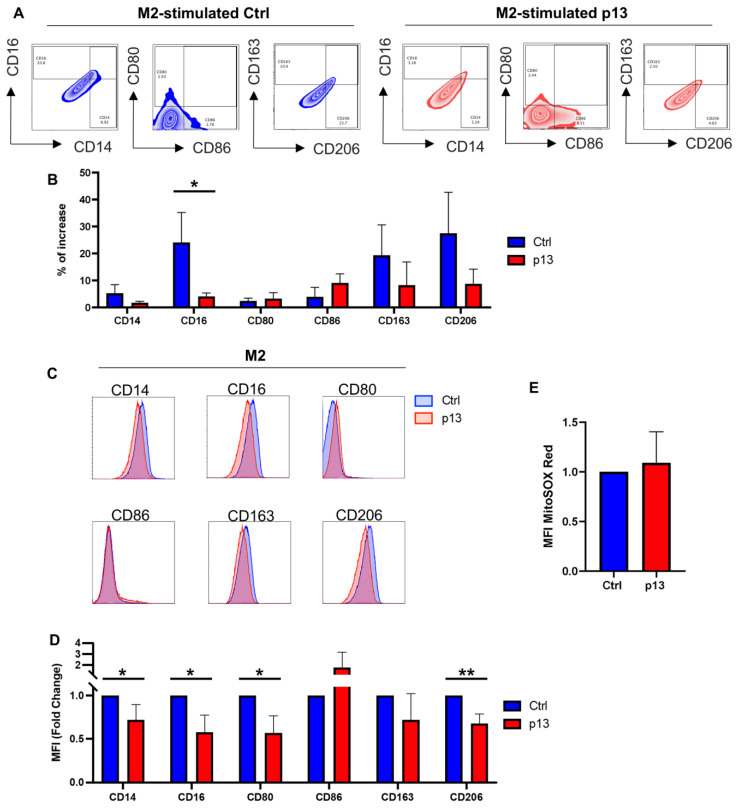
Role of p13 in M2 macrophage polarization. (**A**) THP-p13 and THP-Ctrl cells were treated with PMA at a final concentration of 10 ng/mL for 24 h. Cells were washed gently with medium, and appropriate stimulation was added for 48 h. M2 stimuli: IL-4 (25 ng/mL) and IL-13 (25 ng/mL). After 48 h, cells were collected and stained for CD14, CD16, CD80, CD86, CD163, and CD206 surface markers. Viability dye was also included. Cells were gated by size, single and live cells. THP-p13 cells were also gated for GFP. The percentage of increased CD14 and CD16, CD80 and CD86, and CD163 and CD206 positive cells following stimulation was graphed. (**B**) The percentage of increased frequency of CD14, CD16, CD80, CD86, CD163, and CD206 in THP-p13 compared to THP-Ctrl was graphed. The results of three independent experiments are shown: Ctrl cells are labeled in blue, and p13-expressing cells are labeled in red. (**C**) Representative MFI histograms for CD14, CD16, CD80, CD86, CD163, and CD206 surface markers. Ctrl and p13-expressing cells are colored in blue and red, respectively. (**D**) Fold change in MFI of CD14, CD16, CD80, CD86, CD163, and CD206 surface markers for THP-p13 cells compared to THP-Ctrl is graphed for three independent experiments; Ctrl labeled in blue and p13 labeled in red. (**E**) MitoSOX-based flow cytometry with Red fluorescence was used to measure mitochondrial ROS formation in M2 differentiated THP-p13 or THP-Ctrl cells. The results of three independent experiments were graphed as a fold change. ROS formation was not statistically significant in M2 differentiated THP-p13 compared to THP-Ctrl cells. (**B**,**D**,**E**) Statistical significance was verified with a Student’s *t*-test and reported in the figure. *p*-values are summarized with asterisks, * (*p* ≤ 0.05), ** (*p* ≤ 0.01).

M2 macrophages are distinguished from M1 by their expression of high levels of the mannose receptor CD206 (or MRC1) or the scavenging receptor CD163 and reduced expression of CD80 and CD86. We used PMA stimulation followed by IL-4 and IL-13 to induce M2 macrophage polarization in THP-1 cells. As expected, following stimulation, a clear increase in surface expression of CD163 and CD206 is observed on THP-1 cells compared to the untreated control cells (Figure S5A,B, green). Notably, CD80 and CD86 expression were not affected by M2 stimulation (Figure S5A,B, green). When THP-Ctrl cells were treated with M2 stimuli (PMA/IL-4/IL-13), an increase in the expression of CD163, CD206, and CD16 compared to untreated cells is measured (Figure 4A,B). Likewise, M2 stimulation of THP-p13 cells induces an increase in CD163 and CD206-expressing cells. However, M2 stimulation also induces CD86 on THP-p13 cells. While M2 stimulation increases CD16-expressing THP-13 cells, the percent increase in CD16-expressing cells was significantly lower in THP-p13 compared to THP-Ctrl cells (Figure 4B). Further, a significant reduction in the change in MFI of CD14, CD16, CD80, and CD206 expression was found in THP-p13 compared to THP-Ctrl cells (Figure 4C,D). When ROS activation in M2 differentiated cells is measured, no significant difference in the level of MitoSOX between THP-p13 and THP-Ctrl cells is detected (Figure 4E).

Overall, our data demonstrate that HTLV-1 p13 expression in monocytes affects metabolic activity and disrupts macrophage polarization, altering the induction of surface marker expression in response to external stimuli.

### 3.4. p13 M1 Like Macrophages Induced Migration of CD4^+^ T Cells

**Figure 5:**
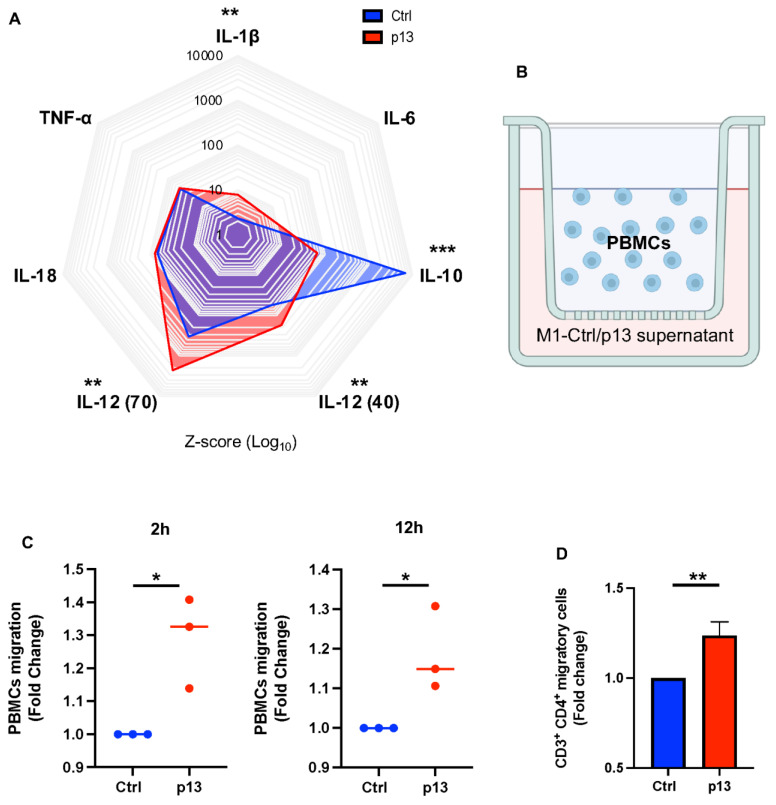
Functional role of M1 p13 cytokine profile. (**A**) Cytokine release of THP-p13 or THP-Ctrl cells following M1 stimulation was measured by Luminex multiplex and graphed as a radial plot (z-score). (**B**) Schematic representation of experimental design. Supernatant from M1 differentiated THP-Ctrl or THP-p13 cells was added to the lower chamber of the trans-well plate. Human PBMCs isolated from 3 different normal donors were plated in the upper trans-well chamber. (**C**) Migration of human PBMCs was measured by cell count at 2 or 12 h following incubation. (**D**) Following migration, cells were collected and stained for CD3^+^ CD4^+^ and viability dye. The frequency of CD3^+^ CD4^+^ cells was used to calculate the total number of CD3^+^ CD4^+^ migrated cells, which was graphed as a fold change. (**A**,**D**) Statistical significance was verified with a Student’s *t*-test and reported in the figure. *p*-values are summarized with asterisks, * (*p* ≤ 0.05), ** (*p* ≤ 0.01), and *** (*p* ≤ 0.001).

M1 macrophages are involved in fighting infection and producing pro-inflammatory cytokines. Therefore, we further characterized the effect of p13 on M1 macrophage function by evaluating cytokine production of PMA/LPS/IFN-γ stimulated (M1-stimulated) THP-Ctrl compared to M1-stimulated THP-p13 cells. Using the Luminex platform, we measured the cell-free supernatant concentration of IL-1β, IL-6, IL-10, IL-12 p40, IL-12 p70, IL-18, and TNF-α. M1-stimulated THP-p13 cells had higher supernatant concentrations of the pro-inflammatory cytokines IL-1β, IL-12 p70, and IL-12 p40 and a lower supernatant concentration of the anti-inflammatory cytokine IL-10 compared to M1-stimulated THP-Ctrl cells (Figure 5A) despite reduced surface marker expression.

Proinflammatory cytokines serve to recruit immune cells to the site of infection [56,57]. Therefore, we tested if the change in the cytokine profile of M1-stimulated THP-p13 cells compared to M1-stimulated THP-Ctrl cells affected cell recruitment. Cell-free supernatants from M1-stimulated THP-p13 or M1-stimulated THP-Ctrl cells were placed in the bottom chamber of trans-well culture plates. Human PBMCs from three independent donors were placed in the upper chamber of the trans-well (Figure 5B). The migration of cells after 2 or 12 h of culture was quantified. A significant increase in the number of cells that migrated across the trans-well was measured at both 2 and 12 h in response to the supernatants from the M1-stimulated p13-expressing cells compared to the M1-stimulated control cells (Figure 5C). Although cytokines in the supernatant of the M1-stimulated THP-p13 cells increased cell migration compared to THP-Ctrl cells, we did not detect a difference in the type of cells that migrated as the frequency of CD4^+^ T cells was unchanged. However, an increased number of migrating CD4^+^ T cells was noted in the supernatant of the M1-stimulated THP-p13 cells compared to the THP-Ctrl cells (Figure 5D).

**Figure 6:**
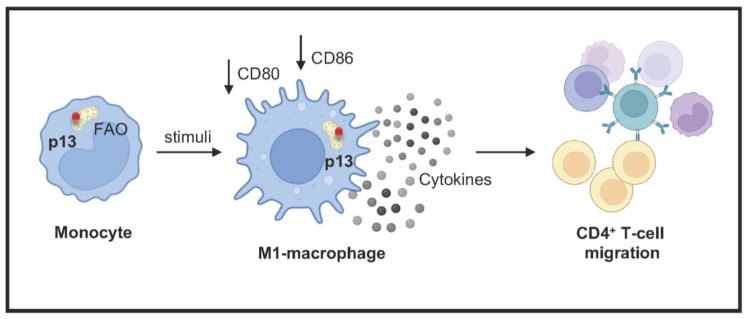
Biological effect of p13 expression in myeloid cells. Schematic representation of the putative role of HTLV-1 p13 protein in monocytes. The p13 protein (red) localizes to the mitochondria, induces metabolic changes, and influences cell differentiation. p13 reduces the expression of surface receptors (CD80 and CD86) affecting M1 macrophage polarization, a critical aspect of antiviral responses, and alters cytokine/chemokine release. This shift in the immune response may be associated in vivo with the recruitment of CD4^+^ T cells, the primary target of HTLV-1, potentially facilitating the spread of infection.

Overall, our results demonstrate that p13 localizes to the mitochondria in monocytes and does not affect either virus proliferation or T-cell response to apoptotic stimuli. However, as summarized in Figure 6, p13 expression in monocytes causes an energetic shift to fatty acid oxidation, reduces surface marker expression in response to stimuli, and induces a distinct cytokine/chemokine profile that favors cell migration. Thus, the effect of p13 on monocyte function may contribute to the immune dysregulation observed in HTLV-1 infection.

## 4. Discussion

HTLV-1 infects and transforms CD4^+^ T lymphocytes, altering cellular differentiation, activation, and survival [58]. In vivo and in vitro studies on p13 function have shown that p13 can induce mitochondrial fragmentation, reduce cell proliferation, and promote apoptosis by ceramide and FasL or glucose deprivation [13,42,45,59]. In addition, in T cells, the viral protein p13 targets the inner mitochondrial membrane, affecting ROS homeostasis and cell turnover [11]. P13 induces ROS production and cell death by enhancing the effects of proapoptotic stimuli in transformed T cells [13,59]. These results suggest that p13 might act as a “viral tumor suppressor” in the context of transformed T cells by reducing proliferation and favoring cell death. Interestingly, the expression of p13 in primary quiescent T cells results in ROS-dependent cellular activation [45]. This dual effect of p13 in primary versus transformed cells is consistent with the rheostat model that connects ROS levels with cell fate [60]. According to this model, gradually higher levels of ROS induce cells into resting, activated, transformed, or apoptotic states [61]. It has been proposed that by targeting mitochondria, p13 favors life-long viral persistence by expanding the pool of infected lymphocytes and eliminating cells that acquire a transformed phenotype [45,59].

Past research mainly investigated the effect of HTLV-1 viral proteins on lymphocytes. However, the viral DNA is also present in myeloid cells in patients, including dendritic cells, monocytes, and macrophages [28,62,63,64,65]. Given the central role of ROS and mitochondrial metabolism in monocyte biology [66], we hypothesized that the viral p13 targets the mitochondria of monocytes, impairing their function and shaping innate immune responses. Supporting this hypothesis, our data in monocytes showed that p13 co-localizes with the mitochondrial resident protein COXIV, a well-established inner membrane mitochondria marker, without affecting cell proliferation [50] and targets mitochondria functions. We used the Seahorse assay to measure the oxygen consumption rate (OCR) in response to modulators of key components of the mitochondrial electron transport chain (ETC) and found that p13-expressing cells have lower maximal respiration capacity. Interestingly, a study of glucose metabolism and oxygen tension on HTLV-1 latency and reactivation in T cells showed that inhibition of glycolysis or mitochondrial ETC suppressed HTLV-1 transcription [67].

In HTLV-1-infected individuals, the frequency of monocytes in the peripheral blood is altered, and their ability to differentiate is impaired. Nascimento et al. [64] observed a decreased frequency of intermediate monocytes (CD16^+^CD14^+^) in HTLV-1 patients compared to healthy donors, while the frequency of classical monocytes (CD16^−^CD14^+^) was not affected. Non-classical monocytes (CD16^+^CD14^−^) were not investigated. Another study of monocyte subpopulations showed an increase in non-classical (CD16^+^CD14^−^) and a decrease in classical monocytes (CD16^−^CD14^+^) monocytes in HTLV-1 patients [28]. Consistent with decreased classical monocytes, Makino et al. found that monocytes isolated from infected individuals display downregulation of CD14 [63], which might be involved in the impaired monocyte differentiation capacity. Interestingly, following macrophage differentiation, p13 monocytic cells significantly decreased CD14 expression (Figure 2D and Figure 4D). Moreover, our data showed that p13 impairs macrophage differentiation, including unpolarized M0 (Figure 2D), classically activated M1 (Figure 3D), and alternatively activated M2 (Figure 4D). In response to viral infection, macrophages typically polarize into the M1 proinflammatory phenotype. M1 macrophages are considered antiviral, while M2 macrophages display an immunosuppressive phenotype. Viruses have evolved strategies to counteract the antiviral responses elicited by M1 and instead take advantage of M2-polarized macrophages for efficient replication [68]. Our analysis notably showed a significant increase in ROS production in M1-stimulated p13-expressing cells compared to M1-stimulated control cells, as well as a robust downregulation of M1 proinflammatory macrophage markers, CD80 and CD86. This is consistent with CD86 downregulation observed in DC-derived monocytes isolated for HTLV-1 patients [63]. These findings underscore that HTLV-1-p13 expression in monocytes affects metabolic activity and disrupts macrophage polarization, particularly antiviral M1, by altering the induction of surface marker expression in response to external stimuli. P13 modulation of mitochondrial ROS and potassium influx has been linked to the alpha-helical domain [12], and in future studies, we plan to determine if this same region is important for modulating p13 function in monocytes.

Monocytes and macrophages are central to orchestrating inflammation during viral infection [30] by releasing immunomodulators that activate innate and adaptive responses [31,32]. The role of myeloid cells in HTLV-1 infection and pathogenesis is not fully understood. However, evidence shows that the cytokine profile of myeloid cells is disrupted in the context of HTLV-1 infection. Monocyte-derived macrophages from HTLV-1-infected individuals release lower levels of IL-10 than macrophages from uninfected donors [69], and in vitro, infected monocytes display an increased level of IL-1β production compared to uninfected cells [70]. Moreover, higher production of IL-12 was noted in monocytes isolated from HAM/TSP patients compared to asymptomatic carriers [71]. Since M1 macrophages are typically activated during viral infection and our data showed that p13 impaired M1 polarization, we further characterized the effect of p13 on M1 macrophage cytokine profiles. Our data showed that M1-stimulated THP-p13 cells exhibited higher supernatant concentrations of IL-1β, IL-12 p70, and IL-12 p40 and a lower level of IL-10, consistent with increased inflammation and with previous reports of HTLV-1 infection [69,71].

Cytokine release during viral infection leads to the recruitment of leukocytes to infected tissue. Previous research showed that macrophage stimulation with the HTLV-1 viral protein, Tax, induces the production and release of well-documented chemotactic immunomodulators (CCL3/MIP-1α, CCL4/MIP-1β, and CCL5/RANTES) [72]. These findings suggested that HTLV-1 might manipulate macrophage functions to favor recruitment of CD4^+^ T cells in HTLV-1 target tissue (Figure 6), such as CNS, skin, and lymphoid organs, during infection and development of HTLV-1-associated diseases (HAM/TSP, infectious dermatitis, and ATLL). Our data showed that M1-p13 macrophages release higher levels of proinflammatory cytokines, which might be involved in immune cell recruitment to the site of infection [62,63]. Here, we show that in response to M1-stimulated p13-expressing cells, there is a significant increase in CD4^+^ T-cell migration. Overall, we observed that p13 expression in monocytes influences their metabolism and function, potentially contributing to the immune dysregulation observed in HTLV-1 infection.

## 5. Conclusions

Our findings highlight the effect of the viral p13 protein on monocyte biology. The localization of p13 to the mitochondria induces metabolic changes and influences cell differentiation. We observed an energetic shift to fatty acid oxidation, reduced surface marker expression in response to stimuli, and changes in cytokine production in monocytes expressing p13. By affecting M1 macrophage polarization, a critical aspect of antiviral responses, p13 reduces the expression of surface receptors (CD80 and CD86) and alters cytokine release. The data suggest that this significant shift in the immune response may lead to the recruitment of CD4^+^ T cells, the primary target of the virus, and potentially facilitate the spread of viral infection and the development of disease.

## Data Availability

The original contributions presented in the study are included in the article/Supplementary Material; further inquiries can be directed to the corresponding authors.

## Supplementary Materials

The following supporting information can be downloaded at: https://www.mdpi.com/article/10.3390/v17040471/s1, Figure S1: P13 localization in HeLa cells; Figure S2: Monocyte proliferation of p13 expressing cells; Figure S3: Staurosporine treatment; Figure S4: Seahorse assay in p13-expressing monocytes; Figure S5: In vitro M1/M2 macrophage polarization.
