# Supplementary material for: HTLV-1 p13 Protein Hijacks Macrophage Polarization and Promotes T-Cell Recruitment
Source: Viruses. 2025 Mar 26;17(4):471. doi: 10.3390/v17040471 (PMC12031607; doi:10.3390/v17040471)
Supplement: Supplementary file 1 [file viruses-17-00471-s001.zip › Supplementary Material.pdf]

Supplementary Material

Figure S1. P13 localization in HeLa cells. (**A**) Immunoblot of HA and GFP expression from total cellular extracts of HeLa cells transfected with retrovirus expressing p13-HA-GFP; empty lentivirus used as a control is also included. β-Actin expression was used as a loading control. (**B**) HeLa cells transfected with empty vector (pBABE) or p13 fused with HA and GFP tag were cytospin and stained with an antibody towards complex IV (COXIV). Scale bar: 10μm.

Figure S2. Monocyte proliferation of p13 expressing cells. (**A**) THP-Ctrl and THP-p13 cells were stained with CellTrace™ Far Red, and MFI was measured by flow cytometry every 24 hours for three days following. A representative experiment is shown. (**B**) THP-Ctrl and THP-p13 cells were stained with CellTrace™ Far Red and treated with increased concentration of Staurosporine (0.01, 0.1, 1 μM). MFI was measured by flow cytometry every 24 hours for three days following treatment to measure cell proliferation. Untreated THP-Ctrl and THP-p13 cells were also included. Statistical significance was verified with Student's T-test. No statistical significance was noted in p13 cells compared to the control.

Figure S3. Staurosporine treatment. (**A**) THP-Ctrl and (**B**) p13-expressing cells following treatment with increased concentration of Staurosporine (0.01, 0.1, 1 μM). Cells were stained with viability dyes for flow cytometry to measure the percentage of live cells at 24 and 48 hours following the treatment.

Figure S4. Seahorse assay in p13-expressing monocytes. Seahorse's extracellular flux analysis measured the oxygen consumption rate in p13-expressing and control cells. (**A,B**) Representative seahorse rate, bar graphs of **(A)** basal respiration, and **(B)** ATP production are shown in the figure. Statistical significance was verified with Student's T-test. No statistical significance was noted in p13 cells compared to the control. (**C**) Seahorse's extracellular flux analysis measured the oxygen consumption rate in p13-expressing and control cells pretreated with etomoxir was graphed. Representative seahorse rate. Statistical significance was verified with Student's T-test. P values are summarized with asterisks, * ($P \leq 0.05$), ** ($P \leq 0.01$), *** ($P \leq 0.001$), and **** ($P \leq 0.0001$).

Figure S5. ***In vitro*** **M1/M2 macrophage polarization.** (**A**) THP-Ctrl cells were treated with PMA at a final concentration of 10ng/ml for 24 hours. Cells were washed gently with medium and appropriate stimulation was added for 48 hours. M1 stimuli: LPS 15ng/ml and IFN-γ 50ng/ml. M2 stimuli: IL-4 25ng/ml and IL-13 25ng/ml. Following 48 hours, cells were collected and stained for CD14, CD16, CD80, CD86, CD163 and CD206 surface markers. Viability dye was also included. Cells were gated by size, single and live. Plots of CD14 and CD16; CD80 and CD86; CD163 and CD206 are graphed. (**B**) The figure shows histograms of CD14, CD16, CD80, CD86, CD163, and CD206 surface markers. Untreated Ctrl, M1, and M2 stimulation are colored in gray, green, and magenta, respectively.
