# Supplementary figures and images for: HTLV-1 p13 Protein Hijacks Macrophage Polarization and Promotes T-Cell Recruitment
Source: Viruses. 2025 Mar 26;17(4):471. doi: 10.3390/v17040471 (PMC12031607; doi:10.3390/v17040471)

**Fig S1**

**A**

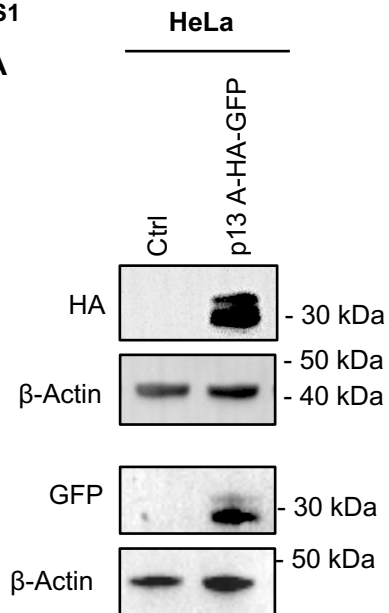

**B**

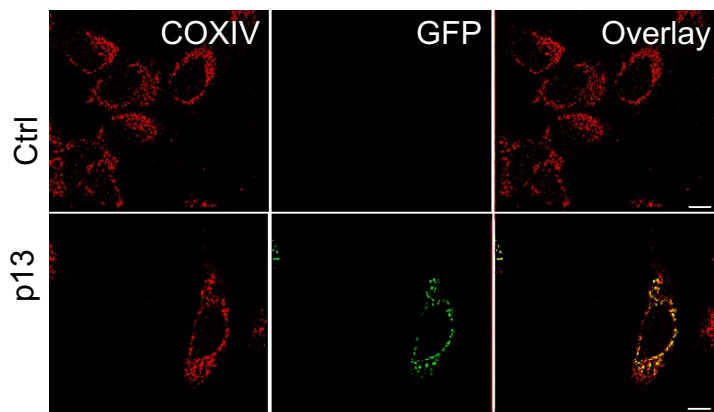

Fig S2

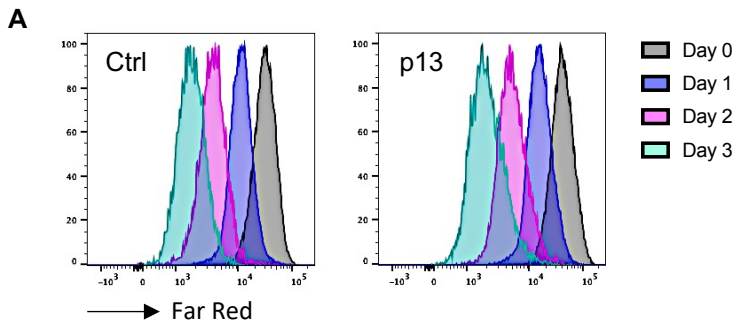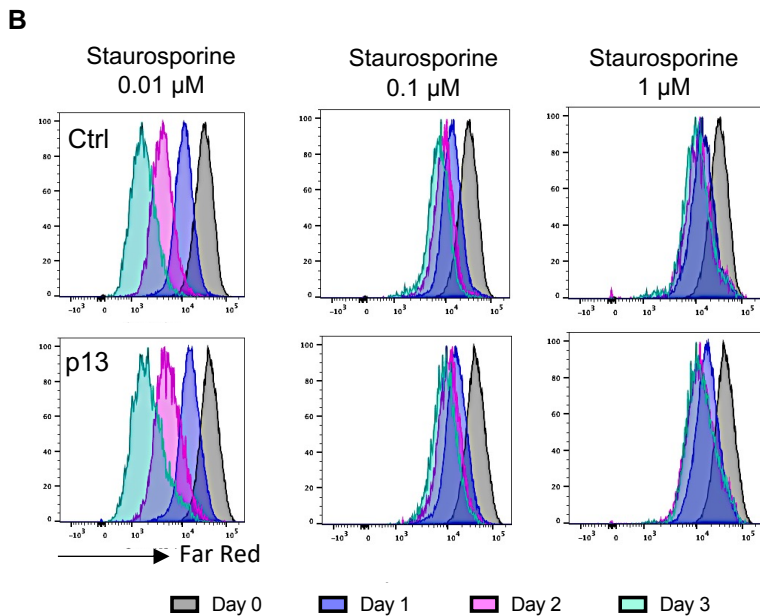

**C**

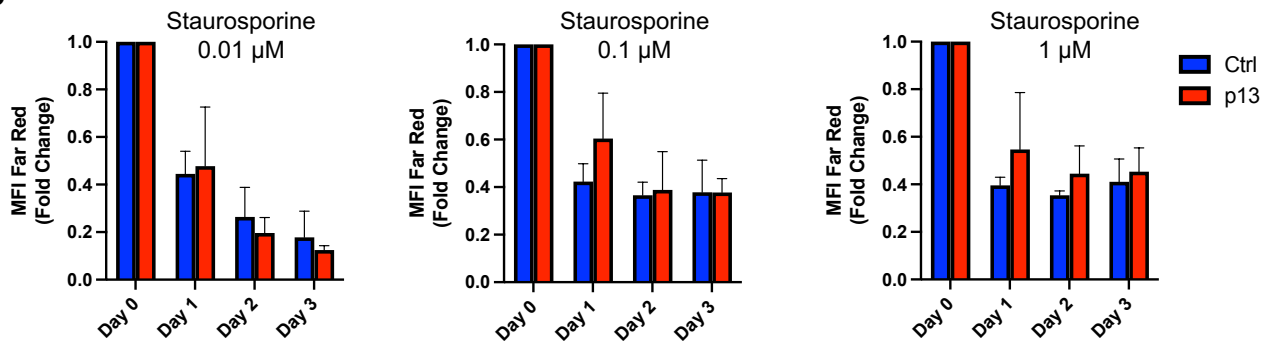

**Ctrl**

**p13**

**A**

**B**

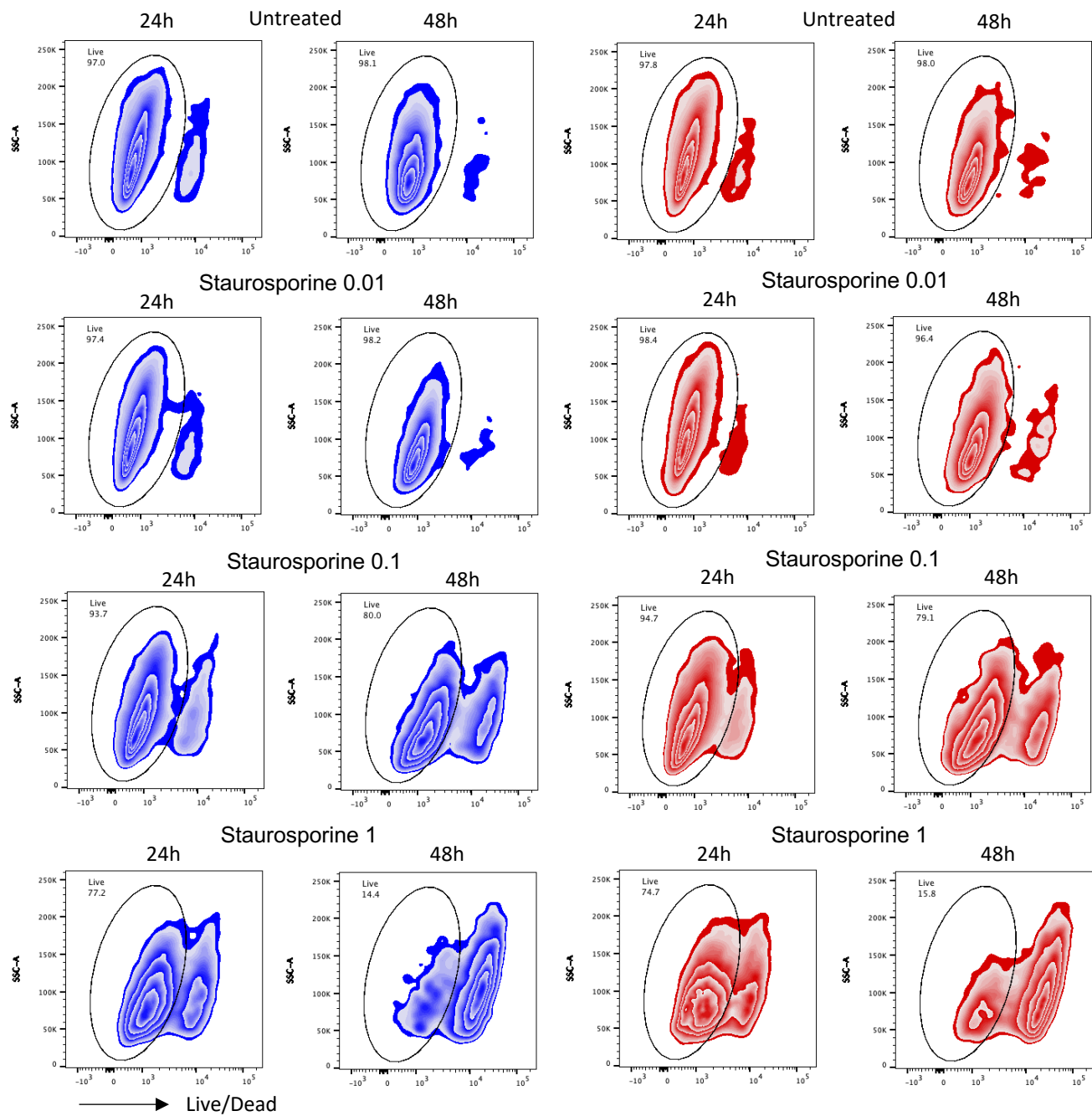

Fig S4

A

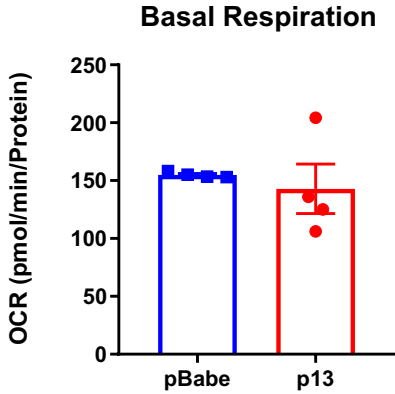

B

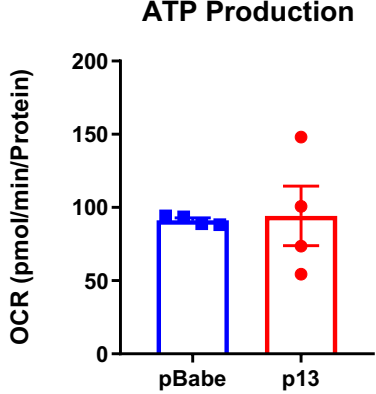

C

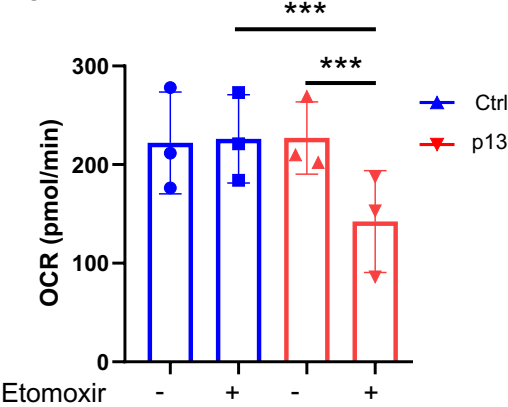

**Fig S5**

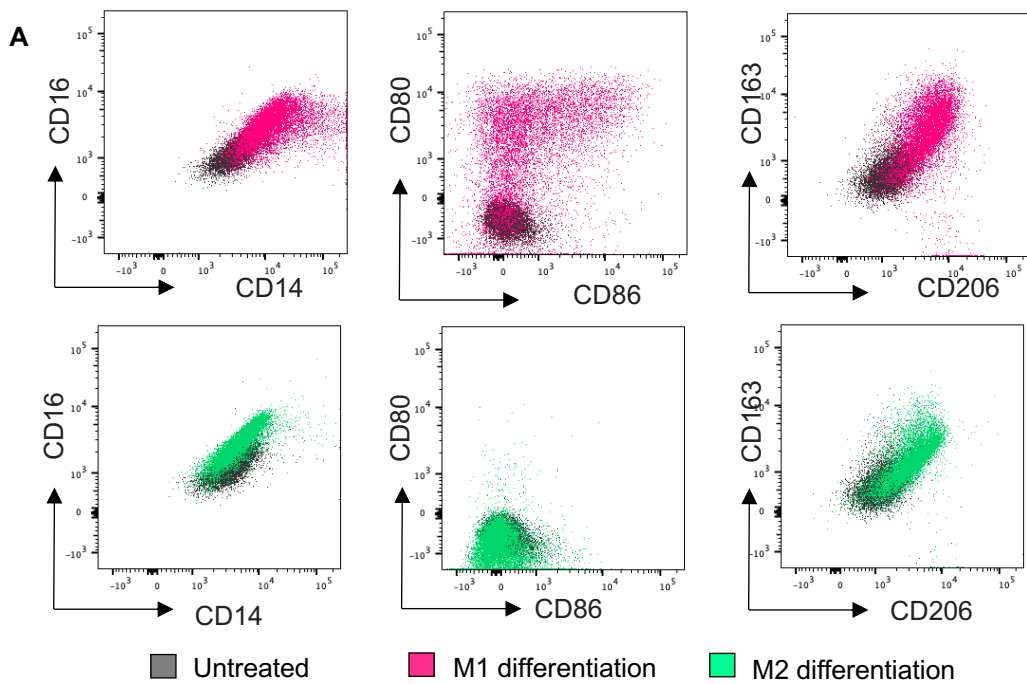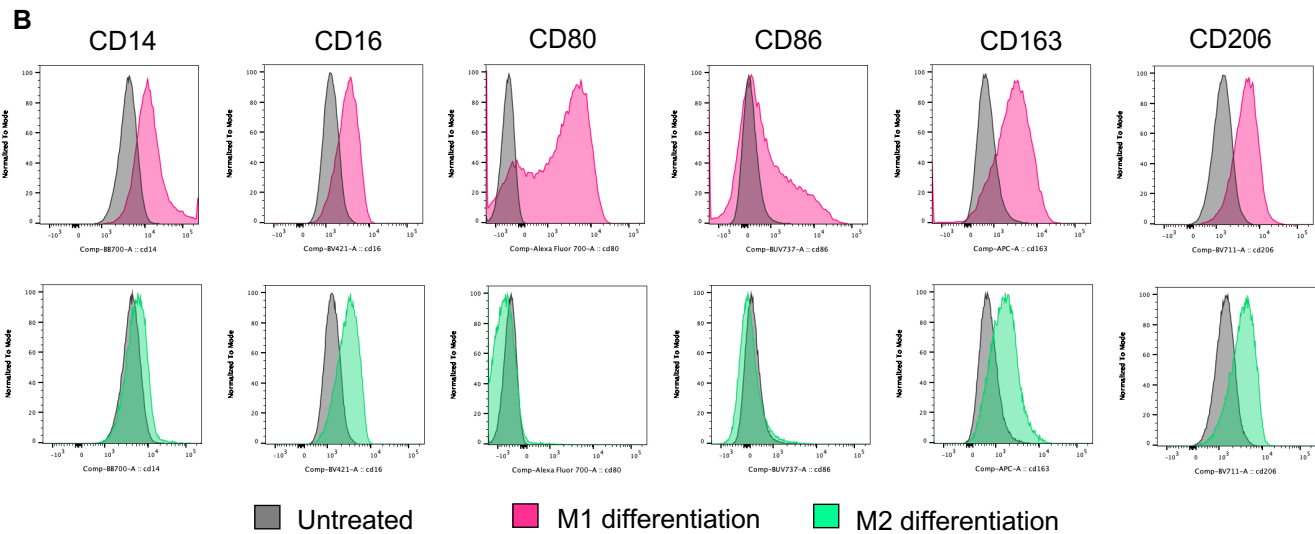

Supplement: Supplementary file 1 [file viruses-17-00471-s001.zip › Supplemental Figures.pdf]
